# Decision tree learning in Neo4j on homogeneous and unconnected graph nodes from biological and clinical datasets

**DOI:** 10.1186/s12911-023-02112-8

**Published:** 2023-03-06

**Authors:** Rahul Mondal, Minh Dung Do, Nasim Uddin Ahmed, Daniel Walke, Daniel Micheel, David Broneske, Gunter Saake, Robert Heyer

**Affiliations:** 1 Faculty of Computer Science, Otto-von-Guericke-University Magdeburg, Universitätsplatz 2, 39106 Magdeburg, Germany; 2 Faculty of Process and Systems Engineering, Otto-von-Guericke-University Magdeburg, Universitätsplatz 2, 39106 Magdeburg, Germany; 3 German Centre for Higher Education Research and Science Studies, Lange Laube 12, 30159 Hannover, Germany; 4 Research Group Databases and Software Engineering, Faculty of Computer Science, Otto-von-Guericke-University Magdeburg, Universitätsplatz 2, 39106 Magdeburg, Germany

**Keywords:** Graph database, Neo4j, Cypher, Decision tree, Java, Python, R

## Abstract

**Background:**

Graph databases enable efficient storage of heterogeneous, highly-interlinked data, such as clinical data. Subsequently, researchers can extract relevant features from these datasets and apply machine learning for diagnosis, biomarker discovery, or understanding pathogenesis.

**Methods:**

To facilitate machine learning and save time for extracting data from the graph database, we developed and optimized Decision Tree Plug-in (DTP) containing 24 procedures to generate and evaluate decision trees directly in the graph database Neo4j on homogeneous and unconnected nodes.

**Results:**

Creation of the decision tree for three clinical datasets directly in the graph database from the nodes required between 0.059 and 0.099 s, while calculating the decision tree with the same algorithm in Java from CSV files took 0.085–0.112 s. Furthermore, our approach was faster than the standard decision tree implementations in R (0.62 s) and equal to Python (0.08 s), also using CSV files as input for small datasets. In addition, we have explored the strengths of DTP by evaluating a large dataset (approx. 250,000 instances) to predict patients with diabetes and compared the performance against algorithms generated by state-of-the-art packages in R and Python. By doing so, we have been able to show competitive results on the performance of Neo4j, in terms of quality of predictions as well as time efficiency. Furthermore, we could show that high body-mass index and high blood pressure are the main risk factors for diabetes.

**Conclusion:**

Overall, our work shows that integrating machine learning into graph databases saves time for additional processes as well as external memory, and could be applied to a variety of use cases, including clinical applications. This provides user with the advantages of high scalability, visualization and complex querying.

## Background

Graph databases enable efficient storage of heterogeneous and highly interlinked data, such as clinical datasets [1]. Usually, clinical data sets comprise the patient information, diagnoses, metadata, and results of different examinations (for instance, simple blood pressure measurements, the latest CT and MRT scans, or high-resolution omics data) that are often graph shaped. Subsequently, researchers can extract relevant features from these datasets and apply machine learning for diagnosis, biomarker discovery, or understanding pathogenesis.

However, data extraction and subsequent machine learning using a standard machine learning toolbox have the additional process of storing data in memory external to the database. Hence, a better workflow would be to apply machine learning directly to the data stored in the graph database.

To show the feasibility of this approach, we apply decision tree learning directly in Neo4j and persist the final tree in Neo4j. Therefore, we have created an open-source Neo4j plugin (Decision Tree Plugin (DTP))^1^, which exposes procedures for decision tree creation and execution on data stored in Neo4j. Thus, the final created decision trees can also be visualized in the Neo4j Browser.

While building DTP, we used three clinical datasets to realize common trends of such data, such as missing values, feature handling and evaluation metrics while generating decision tree algorithms in Neo4j. To assess its efficiency, we have evaluated the accuracy, Matthews Correlation Coefficient^2^ and the computational time of our plugin compared to decision tree functions from Python and R on the datasets. Furthermore, we applied our procedures to a fourth two-log fold larger dataset about diabetes to assess big data performance and evaluate its clinical applicability.

**Fig. 1:**
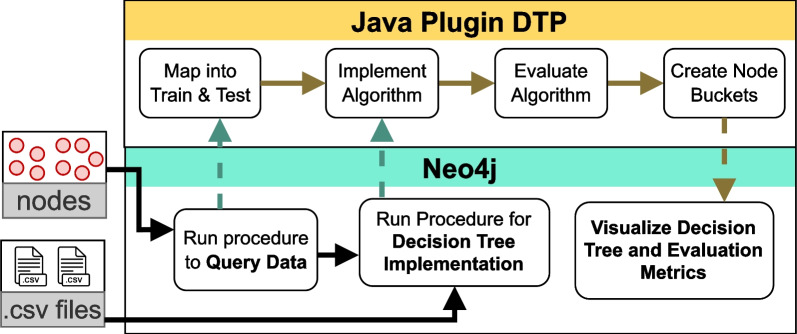
Data flow in the decision tree plug-in (DTP)

In our research on learning algorithms in Neo4j, we contribute the following:

- *DTP* comprises 24 procedures, which can read CSV files, map nodes, split data, generate decision tree using three different splitting criteria (Fig. 1), perform k-fold cross validation, validate the classifier and visualize the decision tree. Moreover, it contains procedures to analyze the features in a dataset, with their respective values of gini index, information gain and/or gain ratio. The novelty of integrating machine learning algorithms is specific to Neo4j and not graph databases in general.
- An extensive comparison of our plugin with state-of-the-art machine learning libraries in Python and R shows that our plugin achieves similar quality of predictions due to the integration of machine learning in the Neo4j graph database. Further, there is only a negligible increase in generation time of algorithms when compared to Python.
- Finally, we have used our plug-in to generate decision tree algorithms on a very large dataset, comprising demographic and pathogenic information of diabetic, borderline diabetic and non-diabetic patients. We used the generated algorithms to gain basic clinical insights on the disease, and prove that this tool could be used for such use cases.

In the following, we will present our research questions, background as well as related work of our study. Thereafter, in the section Methods, we describe the used methodology, our implemented plugin and the used datasets. In the section Results, we evaluate DTP against Java, R and Python decision tree algorithms. Finally in the section Discussion we discuss key aspects of our research and conclude our research by proposing future work in the section Conclusion.

### Research questions

To evaluate the importance and performance of our plug-in, we defined three research questions, which we will answer through the evaluation of our plugin:

#### RQ 1

What is the difference in the quality of predictions for algorithms generated in DTP, when compared against algorithms generated by standard libraries from Python and R?

#### RQ 2

What is the difference in generation time of decision tree algorithms, generated by DTP, compared to standard libraries from Python and R?

#### RQ 3

Could applying decision tree learning on homogeneous and unconnected nodes created from large clinical datasets provide basic clinical insights?

Now we will provide a brief overview on graph databases, decision tree algorithms and a short introduction about diabetes to interpret the clinical background. Afterwards, we will discuss related work on the integration of machine learning in databases.

**Table 1:** Comparison of Database Management Systems [3, 4]. RDBMS, OODBMS and Graph refers to Relational, Object-Oriented and Graph Database Management Systems

	RDBMS	OODBMS	Graph (Neo4j)
Flexibility (lack of schema)	Low	Medium	High
Query language	SQL	Rarely implemented	Cypher
Query performance	High	High	High
Integrity constraints	Yes	Yes	Yes
Level of support	High	Low	High
Ease of programming	High	High	High
Security	High	Low	High
Scalability	Low	High	High

**Fig. 2:**
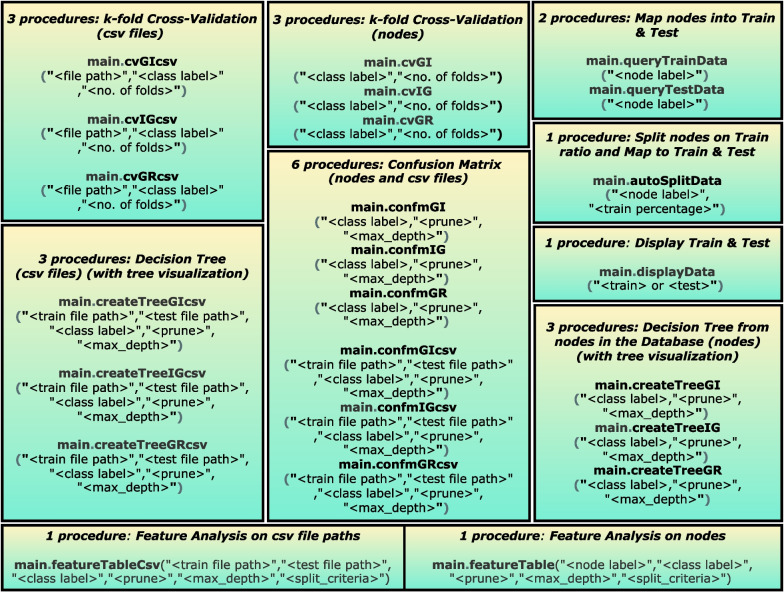
Available procedures in DTP

### Graph database management system: Neo4j

Graph databases like Neo4j (Table 1), equipped with its query language Cypher, are NoSQL databases that store data within a graph structure, enabling flexible queries through interlinked data. The main advantages over relational and object-oriented databases are its flexible data models and schema. Furthermore, graph database are easily scalable, making it ideal to store large datasets and perform read or write operations [5].

In addition to the advantages intrinsic to a graph database, Neo4j offers procedures that handle complex operations [6]. They are implemented in Java [7] and are compiled into a Jar file, which can later be deployed to the database by adding the Jar files into the plugin directory on each individual or clustered server. A procedure can be invoked either as a stand-alone procedure from the application, the command-line or as a part of Cypher statements.

Cypher is a declarative query language, optimized for graphs, and therefore, can be used for describing visual patterns in graphs using ASCII-Art syntax. This makes Cypher queries much simpler and easier to write compared to SQL [8].

There could be three main arguments for using a graph database like Neo4j, to analyse clinical data of any scale:

- *Scalability* Graph databases are scalable [9].
- *Node-edge Structure* This helps in forming and analysing complex relationships. [10]
- *Query Performance* Querying can be versatile in graph databases which helps in analysing complex data structures [9].

### Decision tree algorithms

**Table 2:** Overview about decision tree algorithms grouped by the splitting criteria

	Data types	Missing values	Data splitting
Gini index	Categorical and numerical	Can handle	No restrictions
Information gain	Numerical	Can not handle	No restrictions
Gain ratio	Categorical and numerical	Can handle	Binary

Decision tree algorithms are machine learning algorithms predicting attributes based on tree-like decision rules. In the literature, several implementations of decision tree algorithms are known (Patel et al. [11]), including CART (Breiman et al. [12]), ID3 ( Quinlan [13]), C4.5 (Quinlan [14]). These algorithms can be separated based on their splitting criteria (Table 2) into algorithms using either Gini Index (GI), Information Gain (IG), or Gain Ratio (GR).

In our plug-in, we provide procedures for all three splitting criteria—Gini Index, Information Gain, and Gain Ratio. The generated trees are binary, implying a binary split at a decision node, with one path agreeing to a certain threshold and the other one disagreeing.

We have also provided a parameter to perform pre-pruning on the generated decision trees through our procedures and reduce the size of tree as per user choice. This is useful as it can help to overcome over-fitting of decision trees algorithms and remove noise/outliers from training data [15].

### Diabetes

Diabetes is a group of diseases characterized by hyperglycemia. It results either from defects in insulin secretion, insulin action, or both [16]. In general, diabetes has two etiopathogenetic categories. The first, diabetes type I, is also called insulin-dependent diabetes. This diabetes type is caused by the autoimmune destruction of beta cells and is associated with multiple genetic predispositions and environmental factors that are poorly defined. Diabetes type II, called non-insulin-dependent diabetes, is the more frequent type (90–95 % of all diabetes cases).

The risk of developing type II diabetes increases with age, obesity, hypertension, smoking, and lack of physical activity. It is also more frequent in individuals with hypertension or dyslipidemia (i.e., low HDL cholesterol concentration and high LDL-cholesterol concentration) and is associated with a strong genetic predisposition. This form is often undiagnosed for years because of the gradually developing hyperglycemia. At early stages, hyperglycemia is not severe enough to cause diabetes symptoms [16], however, chronic hyperglycemia can cause dysfunction and failure of different organs, e.g., eyes, kidneys, nerves, and heart. Therefore, diabetes is a potential risk factor for stroke and cardiovascular diseases [17]. Hence, our investigations of the diabetes dataset represent an important real-world use case.

### Related work

While the integration of machine learning algorithms into a graph database (i.e., Neo4j) is novel, both supervised and unsupervised learning algorithms were already applied on the following relational database management systems [18] allowing users to implement learning algorithms directly on the database:

- Amazon Redshift
- Blazing SQL
- Google Cloud Big Query
- IBM DB2 Warehouse
- Kinetica
- Microsoft SQL Server Machine Learning Services
- Oracle Cloud Infrastructure (OCI) Data Science
- Vertica Analytics Platform

Focusing on Neo4j, Max De Marzi et al. [19] created custom procedures in Java to predict for a particular data set whether a student passes an exam. However, these procedures are strictly confined to that dataset and cannot be used for other data sets. Analogously, Michael Hunger et al. [20] as well as Anjana and K. Lavanya et al. [21] showed some steps towards machine learning in Neo4j but provided no universal method.

## Methods

This section presents our applied methods to build and optimize the DTP. Furthermore, we describe the datasets used to evaluate our algorithms. For our study, we selected the graph database Neo4j due to its flexibility and the possibility to extend its functionalities using Java procedures, which are the backbone of the whole DTP infrastructure (Figs. 1,2). Java is one of the most versatile languages and can be currently executed on most operating systems. Therefore, Java is a great language choice to equip a graph database like Neo4j, with several tools for machine learning.

Figure 1 highlights the flow of data between Java and Neo4j when the procedures in DTP are executed, either from CSV files or from homogeneous and unconnected nodes. The DTP data flow (Fig. 1) starts with uploading the input data (CSV files or nodes in Neo4j) and splitting them into train and test data.

The next step is to select among the different splitting criteria (see Section Decision tree algorithms) and run the decision tree implementation to generate a classifier. The learned classifier could be evaluated based on a confusion matrix, accuracy, generation time, and Matthews correlation coefficient and could be applied to new data sets. The resulting nodes and edges of the tree are stored in Neo4j, allowing visualization of the resulting decision tree.

We will now elaborate about the used splitting criteria, the implemented stored procedures and the possibility to visualize the resulting decision tree.

### Procedures of the decision tree plugin

To assess the performance of the different algorithms, splitting criteria and Neo4j, we implemented DTP as a set of Java-based procedures in Neo4j (see Fig. 2). The whole package of DTP is saved as a Jar file which should be copied inside the Neo4j plugins directory of a database. Afterwards, the database should be restarted to make these procedures available, through Cypher queries to generate a decision tree.

These procedures build the tree in Java, returns node and edge buckets to Neo4j for tree visualization and afterwards tests the decision tree with the test data instances. The test data is recursively passed through the tree until it reaches a leaf node, in which case it returns the found class label – the final prediction. To create the confusion matrix (with or without cross validation), the actual labels are compared against the predicted labels to calculate the accuracy, Matthews correlation coefficient, or output the confusion matrix and the time needed to generate the algorithms.

**Table 3:** Computational complexity analysis

Computational complexity	Time	Space
Train complexity	O(dim*nlog*n*)	O(*n*)
Test complexity	O(depth)	O(*n*)

n = number of instances/nodes dim = number of dimensions/variables depth = depth of generated decision tree

In Table 3 we provide a summary of the computational complexity of the Java procedures contained in DTP, using big O notation. For any decision tree procedure, there are two processes involved—training and testing the algorithm. Training complexity is naturally higher as during testing the task is to just traverse the tree, generated during training, where several calculations of splitting criteria are required.

The procedures that can visualize the tree also contain a parameter—“max_depth”, which will limit the depth of the tree to the specified value. It is a pre-pruning mechanism which will stop decision tree generation when the mentioned depth is reached and majority class label is assigned to impure nodes.

Note that with each tree generated from procedures, our plug-in DTP will generate and display the confusion matrix, accuracy, Matthews Correlation Coefficient and generation time of algorithms. The decision tree procedures in DTP can be categorized as follows:

#### Cross validation (without tree visualization)

Six procedures are provided to perform k-fold cross-validation on a single CSV file or a set of nodes. Users can specify the class label and number of folds for every iteration along with CSV file path, exclusively for tree generation from CSV files. It is important to note that there are no tree visualization for cross validation.

#### Decision tree from CSV files (with tree visualization)

These three procedures are used to create a decision tree from plain CSV files, and the resulting tree is stored in Neo4j. To this end, DTP reads 2 CSV files (of train and test data) and detects whether a variable is categorical or numerical. Furthermore, a user-defined class label can be chosen. DTP then recursively calculates the best splits based on the splitting criteria, depending on the chosen algorithm.

The result of the previous step is a bucket of nodes and relationships which represent the tree, stored as a graph in Neo4j for subsequent inference or visualization. The user can specify file paths for training and testing, and the class label . For pre-pruning, user must also set prune = “True” to set the max_depth value of decision tree.

#### Decision tree from graph-shaped data in Neo4j (with tree visualization)

To create a decision tree from homogeneous and unconnected nodes in Neo4j, a user can map two distinct sets of nodes into training and testing, through the procedures. Alternatively, we have created a procedure that allows the user to automatically split the data into train and test sets.

In order to allow generating the decision tree, we implemented three different functions according to the favored splitting criterion. These functions then use the labeled training data and persists the tree nodes and relationships in Neo4j just as the procedures on the CSV files do. The user can specify the class and for pre-pruning, user must also set prune = “True” to set the max_depth value of decision tree.

#### Decision tree without tree visualization (confusion matrix)

A user is also allowed to generate and assess decision tree algorithms without tree visualization, we have implemented 6 procedures – 3 to generate the confusion matrix from CSV files and 3 to generate it from nodes. This is useful in cases where the dataset is large, and the node visualization creates a significant delay in the tree visualization. The user can specify file paths for training and testing and the class label. For pre-pruning, user must also set prune = “True” to set the max_depth value of decision tree.

#### Feature analysis

For further in depth analysis of each feature in datasets, DTP contains procedures to obtain values for gini index, information gain or gain ratio as calculated at every level while the tree is being generated. This will allow user to have an elaborate overview on how each variable affects the generation of tree at each level. The user can specify pruning of tree to a max_depth for these procedures and can be generated from both nodes and/or csv files.

### Data

**Table 4:** Comparison of datasets used in the experiments

	Instances	Variables	Target categories	Class imbalance
Dataset 1	299	13	2	Medium
Dataset 2	48	50	3	Low
Dataset 3	1485	12	2	High
Dataset 4	253,680	22	3	High

To evaluate our decision tree algorithms, we searched for clinical datasets in Kaggle, GitHub, and in research papers. After surveying and investigating several datasets, we selected four datasets about the prediction of heart failures (Dataset 1), inflammatory bowel disease (Dataset 2), classification between flu and COVID-19 (Dataset 3) and prediction of diabetes among patients (Dataset 4):

#### Dataset 1: heart failure prediction

The heart failure dataset by Davide Chicco and Giuseppe Jurman [22] from Kaggle contains 299 patients’ data with 13 demographics variables and has been used to predict survival of patients using machine learning algorithm. Of the 13 variables, 7 are continuous numeric and the rest are categorical, including the class variable. The class variable is the property DEATH_EVENT, where 1 represents death of a patient (96 instances) and 0 (203 instances) their survival.

#### Dataset 2: prediction of inflammatory bowel disease from microbiome

The inflammatory bowel disease dataset by T. Lehmann [23] consists of 2,969 meta-proteins whose presence has been measured among a group of 48 patients of 3 separate groups. All the variables in this dataset are numeric variables. We have considered only the 50 most abundant meta-proteins while training and evaluating the decision tree algorithms. The class variable is Patient Type – C, CD or UC, where C identifies control patients (20 instances), CD for patients with Crohn’s Disease (13 instances) and UC for patients with Ulcerative Colitis (15 instances).

#### Dataset 3: H1N1/COVID-19 classification

The H1N1/COVID-19 dataset was taken from a research article by Li et al. [24] that applied machine learning on a dataset of 1,485 patients with 50 demographic variables. The class variable is Diagnosis – H1N1 (1072 instances) or COVID-19 (413 instances). Since a lot of variables were plainly null, we have reduced the data to 12 variables out of the 50 available to sharpen the results. There are two numeric variables which are continuous and the rest are categorical variables, including the class variable.

#### Dataset 4: diabetes (type II) health indicators

This dataset was taken from Kaggle and was uploaded by Alex Teboul [25] who has cleaned and consolidated the data from the original BRFSS 2015 [26] dataset consisting of data from a survey of patients concerning diabetes. The original dataset was compiled by the Center for Disease Control and Prevention which is the national public health agency of the United States. The cleaned data consists of 253,680 patients with 22 demographic and clinical variables. The class variable is Diabetes_012 consisting of 3 labels – 0 (**213,703 instances**) indicating patient being non-diabetic, 1 (4631 instances) indicating prediabetes or 3 (**35,346 instances**) indicating patient being diagnosed with type 2 diabetes. There are two numeric variables which are continuous, and the rest are categorical variables, including the class label.

### Experimental setup

**Table 5:** Decision tree configurations for experiments 1, 2 and 3: three datasets for each combination of language and splitting criteria

42 algorithms generated	Gini index	Information gain	Gain ratio
R (rpart, RWeka)	3	3	3
Python (sklearn)	NA	3	3
Java	3	3	3
DTP (csv)	3	3	3
DTP (nodes)	3	3	3

For our evaluation, we have compared the performance of the custom java procedures in our plug-in to algorithms generated by the following standard packages in R and python:

- *R* package “rpart” for gini index and information gain and “JWeka” for gain ratio
- *python* package “sklearn” for gini index and information gain. No standard package implementation was found for gain ratio during evaluation.

All the experiments were carried out on a desktop PC with the following specifications:

- *Processor* AMD Ryzen5 3600, 6 cores (3.6 Ghz)
- *Memory* 16 GB of RAM
- *Graphic* NVIDIA GeForce RTX2070 (8 GB)

#### Experiments 1, 2 and 3: building and optimizing DTP

For our experiments, we applied k-fold cross-validation for all the three datasets and all four approaches (Python, R, Java, Neo4j) and evaluated the algorithms. Each decision tree algorithm was regenerated thirty times with the accuracy, Matthews correlation coefficient and generation time averaged out for all the iterations. The number of folds for cross-validation was varied across the datasets, due to the difference in their instance size (see Table 3).

In total, we generated 42 (see Table 5) cross-validated decision tree algorithms for this evaluation in R, Python, Java^3^, DTP (CSV)^4^ and DTP (nodes)^5^ and 2 (Gini Index and Info Gain) in Python. This was due to the unavailability of a generic package for Gain Ratio in Python.

#### Experiment 4: evaluating DTP on a large dataset

To generate and evaluate decision tree algorithms on Dataset 4, we have performed 5-fold cross validation (80 percent data for training and 20 percent for testing) on the whole dataset in R, Python, Java and DTP (csv) and DTP (nodes), totaling to 14 decision tree algorithms, across the mentioned tools.

## Results

**Fig. 3:**
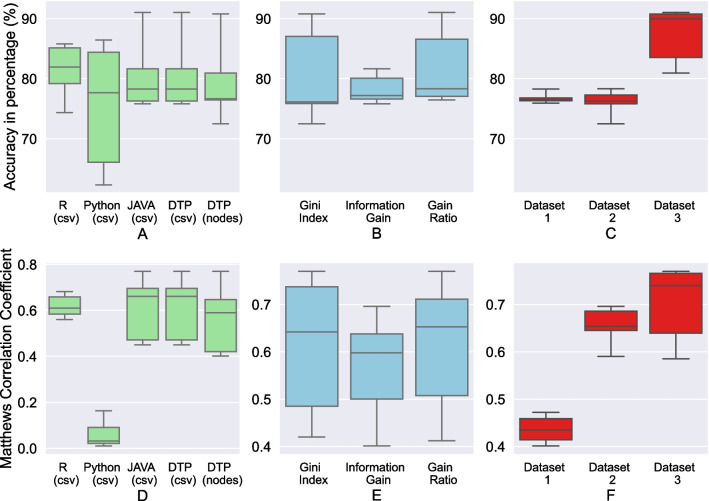
Box Plots—Accuracy and Matthews Correlation Coefficient of the algorithms: **A**, **D** for different tools including DTP, **B**, **E** for different splitting criteria in DTP, and **C**, **F** for the datasets 1-3 in DTP

**Fig. 4:**
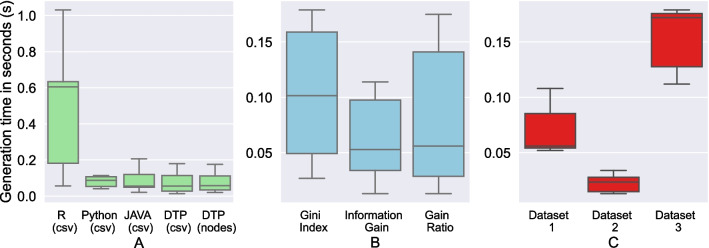
Box Plots—Generation Time of the Decision Tree Algorithms: **A** for different tools including DTP, **B** for different splitting criteria in DTP, and **C** for the datasets 1-3 in DTP

**Fig. 5:**
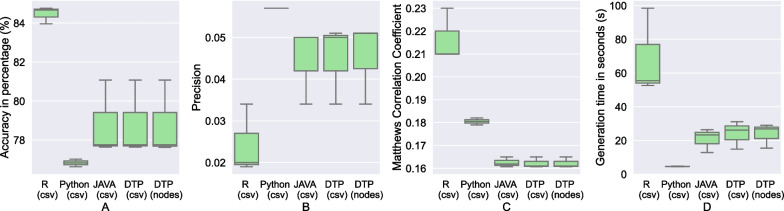
Box Plots—Evaluation of the diabetes dataset(Dataset 4), across different tools: **A** accuracy, **B** precision, **C** Matthews Correlation Coefficient, and **D** generation time

In the following, we examine the performance of DTP in Neo4j compared to standard machine learning algorithms in R and Python at first for the small datasets 1-3 and afterwards for the big dataset 4.

### Experiment 1: prediction performance comparison

In our first experiment, we assessed whether the different implementations and thus data accesses impact the accuracy and Matthews correlation coefficient (MCC). To compare the accuracy distribution visually, we visualized box plots in Fig. 3. The results show that all algorithms act in the same accuracy and MCC range.

While our DTP trained on data in Neo4j and from CSV files is on par with the Java implementation, it is clearly more stable than the Python decision tree. Considering the different splitting algorithms, gain ratio is the best metric providing the best median value. Low MCC for Python (Fig. 3A) was caused by overfitted decision trees along with class imbalance in Dataset 3 (Table 4).

### Experiment 2: computational time comparison

Next, we evaluate the generation time of DTP-Neo4j-Plugin for the three test datasets against the implementations from R and Python in Fig. 4. The Neo4j integration had a positive impact on the generation time. The decision tree learning from data inside Neo4j is the fastest and most stable approach. In contrast, R had a big deviation in generation time when loading data from CSV files. A more in-depth investigation has shown that this is due to a lot of data shuffling inside R, which creates a considerable overhead. The investi- gation of the splitting algorithms shows that gain ratio provides the fastest generation times.

The high performance of python, in terms of low generation time, could be attributed to the package sklearn, part of which was written in C and C++, which are extremely fast at compiling. [27]

**Fig. 6:**
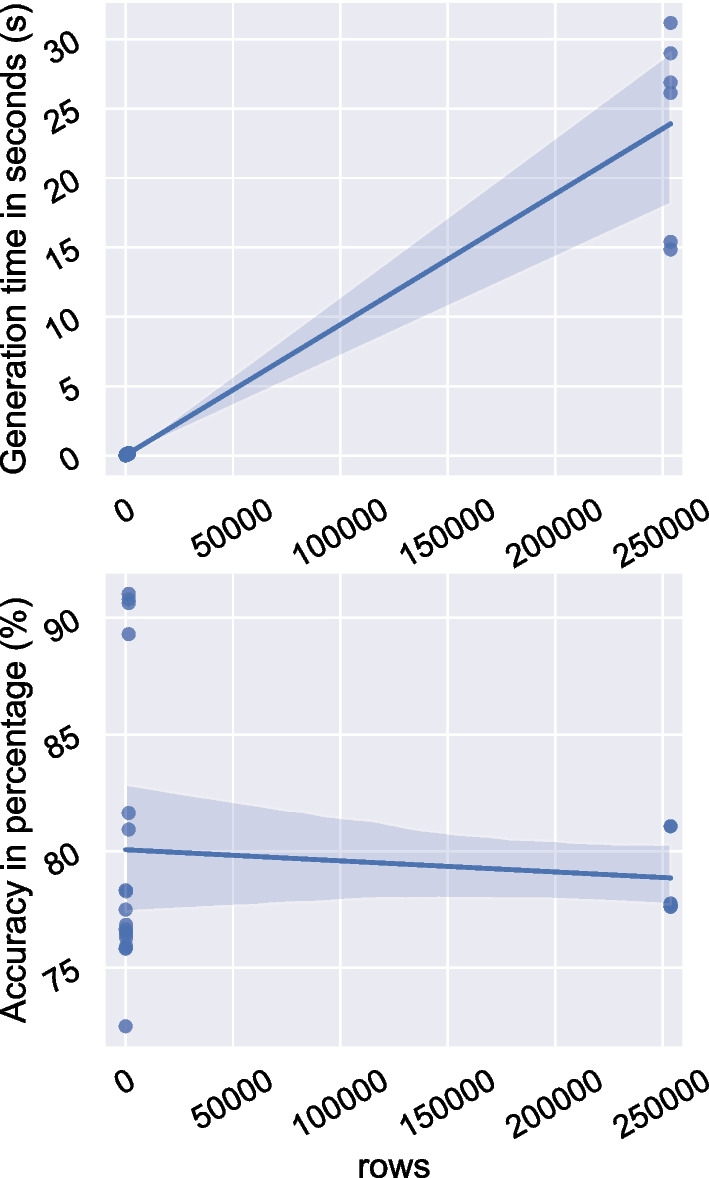
Scatter Plots with Line of Regression—To interpolate the effect of instance size (rows/nodes) on generation time and accuracy of algorithms generated by DTP for all the 4 datasets (Dataset 1, 2, 3 and 4)

### Experiment 3: impact of dataset characteristics

**Fig. 7:**
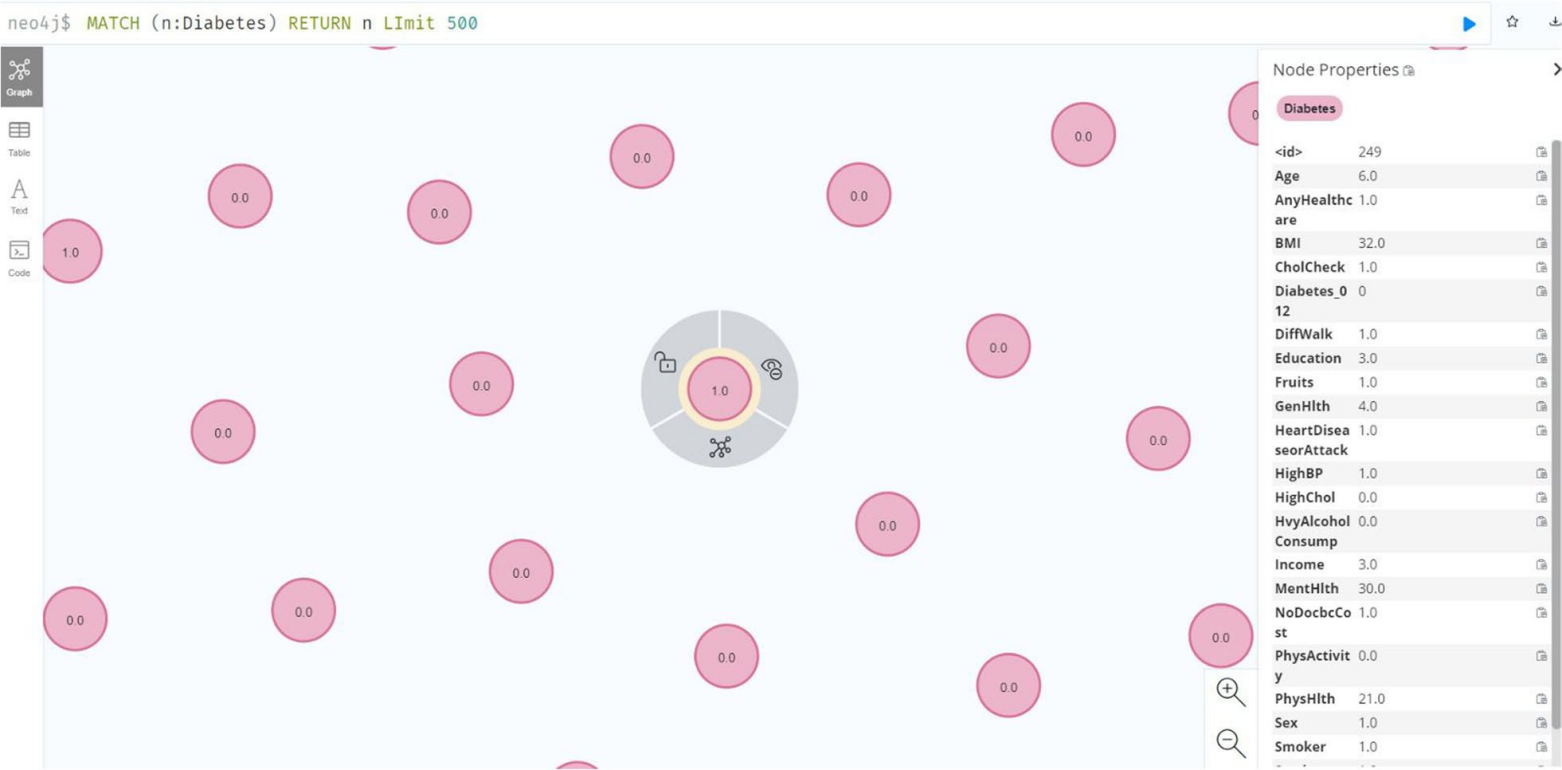
Dataset 4 uploaded as homogeneous and unconnected nodes in Neo4j

Figure 4C (with reference to Table 4) shows that the generation time is directly proportional to the number of instances used while training the algorithm. It might seem that the generation time is directly proportional to accuracy as well which can be explained through a causal link – a higher number of training instances takes up a higher generation time and provides higher accuracy as well, which is quite understandable, since a well-trained algorithm would provide better accuracy.

A regression plot for all algorithms generated on the 4 datasets in DTP is shown in Fig. 6. This is discussed briefly in the next section.

### Experiment 4: evaluation of algorithms generated on dataset 4 to predict diabetes in patients

In this experiment (Fig. 5), we evaluated the different tools while running on the large Dataset 4. Note that the differences in values are quite insignificant with respect to quality of predictions, while there are noticeable differences in the generation time of the algorithms. Python’s package sklearn provides consistently fast performance for Gini Index and Information Gain, regardless of the size of dataset.

To calculate the precision of predictions from Dataset 4, we assumed binary classification while assigning “True Positive” and “True Negative” labels to values in the 3*3 confusion matrix. Hence, the precision values highlight the relevance of the predictions in distinguishing between people with no diabetes (class label = 0) vs. people with borderline and confirmed diabetes (class label = 1 and 2). There was insignificant difference between precision among the different tools.

## Discussion

### Performance of DTP procedures

With slight differences in the experimental results, it is safe to say that DTP performs quite similarly with packages in Python and R, in terms of prediction quality as well as generation time of algorithms. The primary barrier in predictive modelling on large data, such as decision tree classification, is a significant drop in the quality of predictions. Without pruning mechanisms and hyper-parameter tuning, large trees have led to over-fitting and randomness in classification.

Having algorithms generated on 4 datasets of varying size has shown that DTP reflects the scalability of a graph database. We can see in Fig. 6 a slight increase in generation time in seconds while maintaining accuracy over a high range of instance size.

### Neo4j Visualization

All the data used in this research were uploaded as homogeneous and unconnected nodes in Neo4j database for decision tree generation, as shown in Fig. 7, which is a visualization of the patients’ data in the dataset 4. All the variables were mapped as node features, with each node representing a patient.

**Fig. 8:**
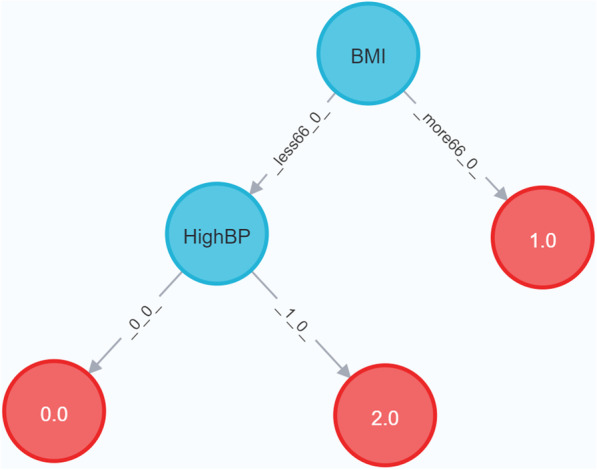
Decision Tree for Dataset 4 (split = gain ratio) The red nodes represent the leaf nodes indicating diagnosis of diabetes (2), borderline (1) or no diabetes (0) in a patient, while the blue nodes are the decision nodes. Note that, this tree was generated on a subset of dataset 4 after the class imbalance was handled. There were 13893 instances (4631 for each class label) and 22 variables. The tree has been pruned to max_depth = 2

Apart from the associated convenient methods for data splitting and validation of the classifier, Neo4j allows for an intuitive visualization of the decision tree as shown in Fig. 8. It stores the final decision tree as a set of nodes and its relationship, which can be stored in Neo4J, queried and reused for classification of further data. The visual interface of Neo4j is quite interactive as a user can move the nodes around, focus on any specific node and also pass a single instance for classification through cypher queries.

### Risk factors for developing diabetes

After generating several decision tree algorithm in DTP and using feature analysis procedures to compare values of gini index, information gain and gain ratio, we could confidently say that the main risk factors for developing pre-Diabetes and Diabetes were high body mass index and high blood pressure (Fig. 8). This fits to the general statements of the center of disease and control and prevention^6^ and confirms the potential of using DTP for mining clinical datasets.

### Discussion of research questions

To conclude the evaluation, we will answer the research questions proposed in section Research Questions.

**RQ 1**—What is the difference in the quality of predictions for algorithms generated in DTP, when compared against algorithms generated by standard libraries from Python and R?

**Answer**—DTP revealed slightly less accuracy as R and Python, but better MCC coefficients. The differences were due to overfitting and could be further diminished by pruning and hyper-parameter tuning.

**RQ 2**—What is the difference in generation time of decision tree algorithms, generated by DTP, compared to standard libraries from Python and R?

**Answer**—DTP required less computational time than R or python for small datasets, but more time than python for large data sets.

**RQ 3**—Could applying decision tree learning on homogeneous and unconnected nodes created from large clinical datasets provide basic clinical insights?

**Answer**—This is clearly possible as we have been able to analyze Dataset 4 by generating a decision tree in DTP and state the following—“High body-mass index and blood pressure are primary risk factors for developing diabetes.”

## Conclusion

In this paper, we investigated the feasibility of integrating decision tree learning, applied directly on graph-shaped clinical data in Neo4j. To this end, we implemented a plugin for Neo4j as a set of stored Java procedures that allow to train and persist a decision tree in Neo4j. When it comes to incorporating cross-platform tools, Java packages, though time-consuming to create and refine, can outperform other platforms in accuracy and computational efficiency. As such, also our Neo4j plugin DTP has reached similar performance compared to Python and R.

Being written in Java, DTP could be easily extended and further optimized. However, at the time of compiling this manuscript, python drivers for Neo4j has been released, and we believe integrating a high performance programming language like python might possibly increase the performance of learning algorithms in Neo4j.

The node-edge view of data and the classifier will facilitate the data analysis. To further improve the data mining, researchers can enrich the graph by further biological and clinical metadata. In numerous ways, researchers are trying to incorporate the strengths of graph database into predictive modelling. With the additional advantage of interactive visualization, researchers are turning to graph data for their research to create novel implementations of traditional statistical and machine learning algorithms.

Neo4j is also helpful in the visualization and analyses of clinical data. The node-edge structure is quite effective to visualize patients with several variables which shows promise for further research. This work motivated us to continue research to incorporate all forms of learning algorithms in graph databases—unsupervised, supervised, semi-supervised and representation learning. One such approach to perform link prediction on scholarly data, in Neo4j, has been performed by Sobhgol et al. [28] which has provided promising results in accuracy, even more so in the computational efficiency, similar to our results in DTP.

Proposed scope of future research could be integration of learning algorithms using python drivers, post-pruning mechanisms on DTP, implementation of decision tree classification on homogeneous and connected nodes, and/or heterogeneous nodes (for both connected and unconnected) in Neo4j.

## Data Availability

The dataset 1 (Heart Failure Prediction), analysed during this study, is available in the Kaggle repository, https://www.kaggle.com/andrewmvd/heart-failure-clinical-data. The dataset 2 (Prediction of Inflammatory Bowel Disease from Microbiome), analysed during this study, is available in the GitHub repository, https://github.com/clumsyspeedboat/Decision-Tree-Neo4j/blob/main/Dataset%202%20-%20Metaprotein/Dataset_Metaprotein.csv. The dataset 3 (H1N1 COVID19 Classification), analysed during this study, is available in the GitHub repository, https://github.com/yoshihiko1218/COVID19ML. The dataset 4 (Diabetes Health Indicators), analysed during this study, is available in the Kaggle repository, https://www.kaggle.com/alexteboul/diabetes-health-indicators-dataset. The source codes of Decision Tree Plug-in (DTP) are available at, https://github.com/clumsyspeedboat/Decision-Tree-Neo4j

## Footnotes

1 For data and source codes, visit https://github.com/clumsyspeedboat/Decision-Tree-Neo4j

2 Matthews correlation coefficient is a measure of the quality of binary and multi-class classifications which tends to be more reliable than accuracy or f1 score [2].

3 Java refers to the custom built Java procedures working in the backend of DTP, for which, Java, DTP(csv) and DTP(nodes) tend to display similar performance in our experiments.

4 DTP (csv) refers to decision tree algorithms generated from CSV file paths in Neo4j

5 DTP (nodes) refers to algorithms generated from nodes in Neo4j.

6 https://www.cdc.gov/diabetes/basics/risk-factors.html, retrieved 16.02.2022
